# Discovery and validation of key genes and potential mechanisms linked to endothelial cell senescence and carbohydrate metabolism in recurrent spontaneous abortion

**DOI:** 10.1515/med-2026-1464

**Published:** 2026-06-24

**Authors:** Lin Chen, Yu Shao, Xianshun Pan, Ting Lai

**Affiliations:** Department of Gynecology, Guiyang Maternal and Child Health Care Hospital, Guiyang Children’s Hospital, Guiyang, Guizhou, China

**Keywords:** recurrent spontaneous abortion, carbohydrate metabolism, endothelial cell senescence, BDH1, PIK3C2G

## Abstract

**Objectives:**

Recurrent spontaneous abortion (RSA) is a major pregnancy complication with largely unknown causes. Carbohydrate metabolism (CM) disorders can affect the progression of endothelial cell senescence (ES), thereby leading to placental dysfunction. However, research on the interplay between CM and ES in RSA is limited. This study aims to identify key genes at the intersection of CM- and ES-related pathways in RSA via various bioinformatics methods.

**Methods:**

Datasets GSE165004 (training) and GSE26787 (validation) were downloaded from the GEO database. ES- and CM-related genes were obtained from previous literature. Key genes were obtained through WGCNA, two machine learning algorithms, gene expression analysis in the training and validation datasets, and RT-qPCR analysis. A comprehensive evaluation was conducted to assess the diagnostic potential of key genes.

**Results:**

Two genes (BDH1, PIK3C2G) were recognized as key genes in RSA, which were upregulated in RSA. Immune infiltration analysis revealed that the key genes were negatively correlated with three distinct immune cell types. Both genes are linked to the cytokine-cytokine receptor interaction pathway. They were predicted to interact with two transcription factors (FOXL1 and YY1) and several chemicals.

**Conclusions:**

This study identifies BDH1 and PIK3C2G as candidate genes associated with CM and ES-related pathways in RSA.

## Introduction

Recurrent spontaneous abortion (RSA), also known as recurrent miscarriages, refers to two or more pregnancy losses with the same partner before the 28th gestational week, affecting approximately 2–5 % of couples of childbearing age [1]. Genetics, immune system status, endocrine conditions, infectious agents, and anatomical abnormalities all contribute to the complexity of RSA [2]. Miscarriages occur most commonly during the first 12 weeks of pregnancy, accounting for about 80 % of all miscarriages. More than half of early miscarriages are associated with genetic defects in the embryo itself [3]. With the development of genomic and molecular biology techniques, some genetic factors have been identified as associated with RSA [4]. Current targeted treatments for specific etiologies, such as the use of aspirin, heparin, and other drugs during early pregnancy and surgical intervention for uterine anomalies, have improved pregnancy outcomes for some RSA patients [5]. Nevertheless, approximately 50 % of RSA cases have an unknown etiology [6]. Therefore, exploring novel biomarkers and better understanding the molecular biology are pivotal for early management of RSA.

Under long-term stress, endothelial cells gradually lose their proliferative capacity and functional state, manifested as cell cycle arrest, increased apoptosis, enhanced inflammatory factor production, and dysfunction of autophagy, commonly known as senescence [7], 8]. During senescence, endothelial cells undergo morphological and functional changes [7]. Endothelial cell senescence (ES) is a complex biological process that involves multiple mechanisms and signaling pathways, including oxidative stress, cell cycle regulation, and inflammatory response [9]. Evidence indicates that ES can increase the risk of miscarriage [10]. Carbohydrate metabolism (CM) is a key physiological process by which the body converts carbohydrates into energy, primarily involving glycolysis, gluconeogenesis, glycogen synthesis and degradation, as well as multiple metabolic pathways such as the pentose phosphate pathway [10]. In endothelial cells, pyruvate dehydrogenase kinase inhibits the shift from glycolysis to oxidative metabolism, which is crucial for maintaining cellular metabolic balance [11]. Specifically, pyruvate dehydrogenase kinase inhibits pyruvate dehydrogenase activity by phosphorylating it, thereby blocking the conversion of pyruvate to the tricarboxylic acid cycle, leading cells to rely on glycolysis for energy [12]. This metabolic reprogramming not only affects the cell’s energy status but may also promote the senescence process in endothelial cells [12]. A hyperglycemic environment accelerates the senescence of venous endothelial cells; the decline in cellular function is manifested by excessive inflammatory responses, abnormal cell adhesion, impaired angiogenesis, and problematic cellular proliferation. Pregnant women experiencing these symptoms may face pregnancy complications that ultimately lead to adverse pregnancy outcomes [13]. Understanding the potential roles of ES and CM in RSA could offer a new perspective on pathological mechanisms of RSA and establish a theoretical basis for developing new therapeutic strategies.

Despite the known links between CM disorders and ES in placental dysfunction, their specific interplay and key regulatory genes in RSA remain largely unexplored. We hypothesized that genes at the intersection of ES- and CM-related pathways contribute to RSA pathogenesis by mediating downstream pathways. Therefore, this study aimed to identify critical genes associated with ES and CM pathways in RSA using integrated bioinformatics approaches, including WGCNA and machine learning algorithms, and to evaluate their immune correlations and regulatory networks.

## Materials and methods

### Data source

The datasets GSE165004 and GSE26787 were sourced from the Gene Expression Omnibus (GEO) database (https://www.ncbi.nlm.nih.gov/geo/), a comprehensive public repository housing extensive data on various diseases. GSE165004 (GPL16699 platform), comprising endometrial tissue samples from 24 RSA patients and 24 healthy fertile women [14], was used as a training set. GSE26787 (GPL570 platform), containing endometrial samples from five RSA patients and five healthy fertile women [15], was used as an external validation set. A total of 102 ES-related genes (ESRGs) and 355 CM-related genes (CMRGs) were obtained from the previous literature [16], 17], detailed in Supplementary Tables S1 and S2, respectively.

### Differential expression analysis of RSA

The “Limma” R package (version 3.54.0) [18] was used for differential expression analysis between RSA and control samples in the GSE165004 dataset. The raw p-values were adjusted for multiple comparisons using the Benjamini-Hochberg method to control the false discovery rate (FDR). Differentially expressed genes (DEGs) were identified with the following criteria: |log2Fold Change|>0.5 and p<0.05, and were visualized by the volcano plot using the “ggplot” package (v3.5.1) [19]. A heatmap was generated for the top 10 DEGs using the “ComplexHeatmap” toolkit (v2.14.0) [20].

### Weighted gene co-expression network analysis (WGCNA) of key module genes associated with ES and RSA

In the GSE165004 dataset, the expression matrix for the 102 ESRGs was extracted to perform single-sample gene set enrichment analysis (ssGSEA) using the “GSVA” package (version 1.50.0) [21] with default parameters, yielding enrichment scores for each sample. These ssGSEA scores served as the trait of interest (representing ESRG activity) in subsequent analysis.

Separately, the “WGCNA” R package (version 1.71) [22] was applied to the full gene expression matrix of the GSE165004 dataset (comprising all genes after standard preprocessing, without additional filtering beyond the dataset’s normalization)to identify co-expression modules highly associated with ESRGs’ enrichment scores. Specifically, a clustering analysis was conducted on the samples, followed by removing outliers to enhance the reliability and accuracy of the analysis. An appropriate soft threshold was selected as the lowest power for which the scale-free topology fit index (R^2^) reached 0.85. Using the selected soft threshold, a weighted adjacency matrix is constructed, representing the connection strengths between all pairs of genes, and subsequently, the adjacency matrix was converted into a topological overlap matrix. Modules were identified using the dynamic shear tree algorithm, with a minimum module size of 100 genes and a merge cut height of 0.25. The correlation between module eigengenes and ESRGs’ ssGSEA enrichment scores was analyzed by Pearson correlation analysis, with significant correlations defined as p<0.05. The most critical modules depend on the highest positive and negative correlations. Key genes within these modules were identified as those with module membership (MM)>0.6 and gene significance (GS)>0.4.

### Screening and functional enrichment analysis of candidate genes

To identify genes that displayed differential expression in RSA and were associated with both ES and CM, three sets of genes were intersected, namely DEGs, key module genes linked to ES, and CMRGs. A Venn diagram was plotted to show the intersection results using the “ggVennDiagram” package (v 1.7.3) [22]. The overlapping genes were selected as candidate genes. To explore the functional roles of the candidate genes, KEGG and Gene Ontology (GO) enrichment analyses were carried out using the R package “clusterProfiler” (v4.10.1) [23] with p adjust method=“BH”. Items with FDR-adjusted p<0.05 were considered significantly enriched.

### Identification of key genes

The overlapping candidate genes were further analyzed using two advanced machine learning techniques: the least absolute shrinkage and selection operator (LASSO) and support vector machine recursive feature elimination (SVM-RFE). These methods were pivotal in sifting through the data to pinpoint the most relevant genetic indicators. The LASSO regression model was constructed using the “glmnet” package (version 4.1.1) [24] with the best λ value selected from 10-fold cross-validation. The “caret” package (v 6.0.93) [25] was used for the SVM-RFE algorithm. The optimal feature subset was identified as the point at which the model achieved the highest accuracy in 10-fold cross-validation. A Venn diagram was plotted to show the intersection of the algorithms. Afterward, the expression patterns of the overlapping genes were analyzed in the GSE165004 and GSE26787 datasets via the “rstatix” package (v 0.7.2) [26] to perform the Wilcoxon rank-sum test. Genes demonstrating consistent and significant expression trends between RSA and control samples across both datasets were selected as key genes for subsequent analyses.

### Clinical specimen collection

Decidual tissue samples were collected from 30 RSA patients and 30 age-matched women undergoing elective abortion due to unplanned pregnancy at Guiyang Maternal and Child Health Hospital. RSA was defined as two or more spontaneous abortions with the same partner before the 28th gestational week, in accordance with the Chinese expert consensus and previous literature. The mean gestational age at sampling was 7.82 ± 1.34 weeks (range 6–10 weeks) in the RSA group and 8.17 ± 1.45 weeks (range 6–11 weeks) in the control group (p>0.05). The inclusion criteria were: (1) age 20–40 years; (2) a history of ≥2 consecutive spontaneous abortions for RSA patients; no history of spontaneous abortion for control women. Key exclusion criteria included: (1) chromosomal abnormalities; (2) infections; (3) antiphospholipid syndrome (APS) or other autoimmune diseases; (4) uterine anatomical abnormalities; (5) endocrine or metabolic disorders; (6) hormone treatment within three months before surgery; and (7) adverse lifestyle habits (e.g., smoking, alcohol abuse).

### Reverse transcription-quantitative polymerase chain reaction (RT-qPCR) analysis

Total RNA extraction from the decidual tissue samples was conducted using the TRIzol kit (Thermo Fisher Scientific, Shanghai, China). RNA purity and concentration were evaluated using NanoPhotometer N50 (Implen, Germany). RNA integrity was further confirmed by visualization of clear 28S and 18S bands on 1 % agarose gel electrophoresis. RNA was reverse transcribed into cDNA using the SweScript RT II First Strand cDNA Synthesis Kit (Servicebio, Wuhan, China). Real-time qPCR was conducted in technical triplicates for each of the 30 biological replicates per group using Universal SYBR Green qPCR premix (Kermey, Zhengzhou, China) on a QuantStudio™ 5 Real Time PCR System (Thermo Fisher Scientific). A melt curve analysis was performed to confirm the specificity of each PCR amplification, which showed a single sharp peak at the expected Tm. The amplification efficiency (E) of the primers was determined by constructing a standard curve using a 5-fold serial dilution of pooled cDNA. The efficiency was calculated from its slope as E = 10^−1/slope^ − 1, resulting in a range of 95–105 % for the primers. GAPDH was used as the reference gene after confirming its stable expression across all samples using the geNorm software. Gene expression levels were quantified using the 2^−ΔΔCT^ method. The primer sequences are detailed in Supplementary Table S3. MIQE guidelines were followed for all the PCR experiments.

### Immune infiltration analysis

In the GSE165004 dataset, the ssGSEA algorithm within the “GSVA” package was used to quantify immune cell infiltration across tissue samples [27]. The gene sets for 28 distinct immune cell populations were obtained from the previous study [28]. These conserved signatures have been successfully applied to endometrial transcriptomic data in studies of recurrent implantation failure [29], 30]. They are also particularly suitable for decidua due to the inclusion of distinct CD56bright and CD56dim NK cell signatures that reflect the predominant uterine NK phenotype at the maternal-fetal interface. The relative abundance of 28 distinct immune cell populations for each sample was calculated. The abundance differences between groups were assessed using the Wilcoxon rank-sum test; p<0.05 indicated statistical significance. Boxplots were generated using the “ggplot2” package. Spearman correlation analysis was performed using the “psych” R package (v 2.4.3) [31] to investigate the correlation between differentially enriched immune cells and between key genes and differentially enriched immune cells. The absolute value of correlation coefficients (|Cor| > 0.3) and p<0.05 were regarded as significantly correlated.

### Gene set enrichment analysis (GSEA)

The “c2.cp.kegg.v7.5.1.symbols.gmt” gene set was downloaded from the Molecular Signatures Database (MSigDB, https://www.gsea-msigdb.org/gsea/msigdb). Spearman correlation coefficients between each key gene and the remaining genes in the GSE165004 dataset were calculated using the “psych” package. Genes were ranked from highest to lowest according to their correlation coefficients. GSEA was conducted using the “clusterProfiler” package with Benjamini and Hochberg correction, and an adjusted p<0.05 was used for significance. The five most significantly enriched pathways were visualized using the “enrichplot” package.

### Molecular network construction and drug prediction

The JASPAR database (http://www.jaspar.genereg.net) was utilized to identify transcription factors (TFs) that target the key genes. Moreover, potential drugs interacting with the key genes were predicted using the Drug-Gene Interaction Database (DGIdb: https://dgidb.org). The Cytoscape software (v 3.9.1) [32] was employed to visualize the interaction networks between the key genes and TFs/drugs. Based on the results of drug prediction, drugs with high credibility in interacting with biomarkers were selected as key drugs for molecular docking analysis. The 3D structures of the protein encoded by the key genes were obtained from the AlphaFold Protein Structure Database (BDH1: AF-Q02338-F1; PIK3C2G: AF-O75747-F1). The 2D structures of key drugs were retrieved and downloaded from PubChem (https://pubchem.ncbi.nlm.nih.gov/), and then converted into 3D structures in mol3 format using ChemBio2D software. Molecular docking was performed using AutoDock Vina, and the molecular docking results were visualized. A binding energy less than −5 kcal/mol indicated that the drug and the biomarker had good binding ability.

### Statistical analysis

Bioinformatics-related statistical analyses were conducted using R software (v 4.2.2). The Wilcoxon rank-sum test measured the differences between the two groups. Data from RT-qPCR were analyzed using GraphPad Prism software (v 8.0.2) and are presented as the mean ± standard deviation. The difference comparison was conducted using unpaired, two-tailed Student’s *t*-tests. Statistical significance was considered as p<0.05.

**Informed consent:** Written informed consent was obtained from all subjects.

**Ethical approval:** All procedures were conducted with approval from the Institutional Review Board of Guiyang Maternal and Child Health Care Hospital and Guiyang Children’s Hospital (approval number: 2024-112, date: Nov. 12, 2024).

## Results

### Identification of key module genes linked to ES via WGCNA

**Figure 1:**
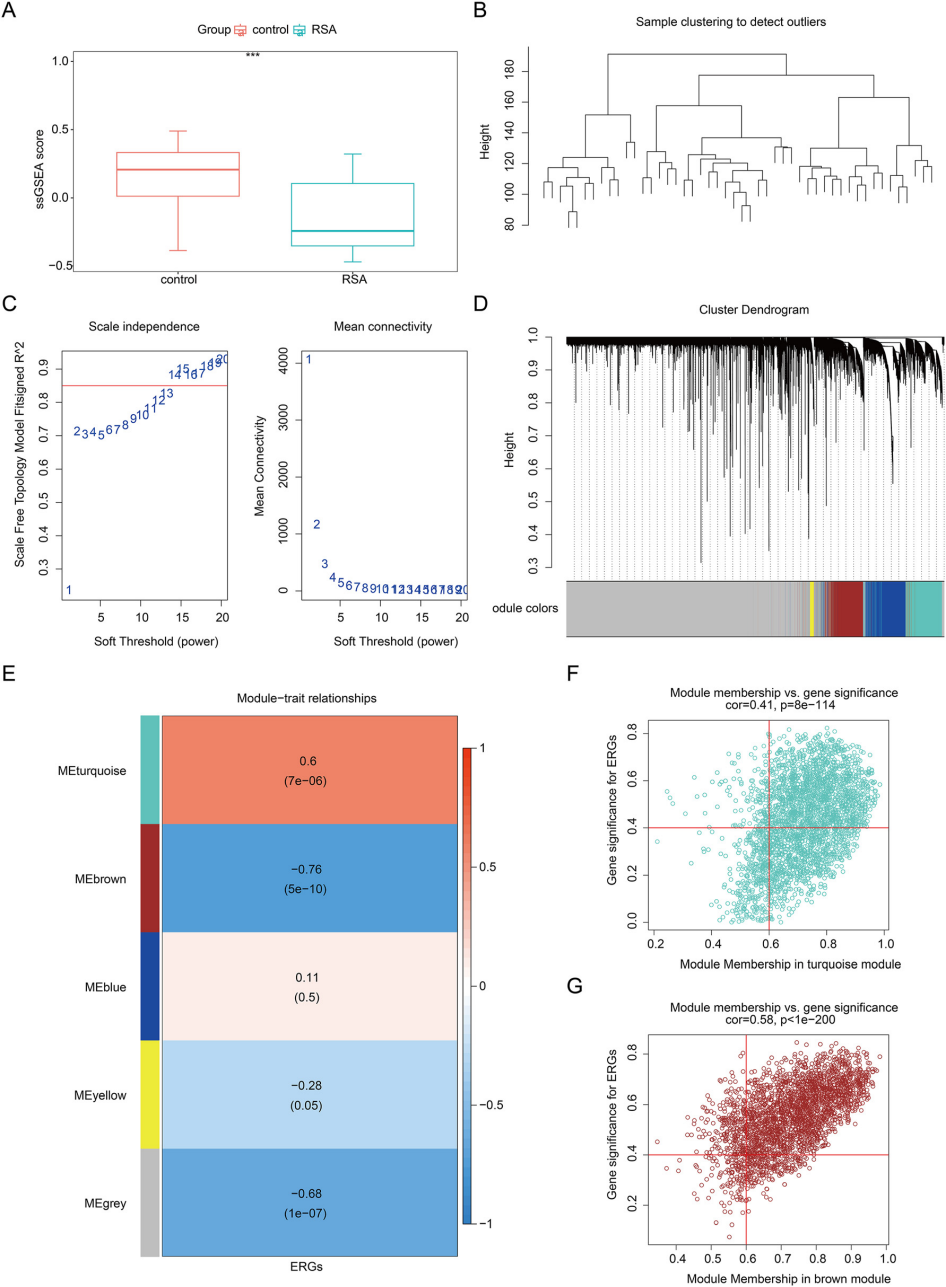
Identification of key module genes linked to ES via WGCNA. (A) Differences in ESRGs’ ssGSEA scores between the RSA and control groups. ***p<0.001. (B) sample clustering dendrogram for detecting outlier samples. (C) Selection of the soft threshold (power). The left panel shows the scale-free topology model fit (R^2^) for different soft thresholds, and the right panel shows the mean connectivity. (D) Cluster dendrogram of gene co-expression modules. The cluster dendrogram of gene co-expression modules illustrates the clustering of genes into different modules. Five major modules are shown, with the gray module representing non-significant genes. (E) Module-trait relationship heatmap. The heatmap displays the correlation between gene co-expression modules and ESRGs scores. (F) The scatter plot shows the correlation between module membership and gene significance for genes in the turquoise module. (G) The scatter plot shows the correlation between module membership and gene significance for genes in the brown module.

As shown by ssGSEA, there was a notable disparity in ESRGs’ activity scores between the two groups. The RSA group had significantly lower values than the control group (p<0.05), suggesting a potential role for ESRGs in RSA progression (Figure 1A). WGCNA revealed that the overall correlation of all samples in the GSE165004 dataset was satisfactory. No outlier samples were detected (Figure 1B). Next, the optimal value for the soft threshold power (β) was determined to be 14 in accordance with the scale-free distribution, ensuring that the network followed a power-law distribution that is essential for the accurate detection of gene co-expression modules (Figure 1C). Five co-expression gene modules were identified, including the meaningless gray module (Figure 1D). Upon analysis, the turquoise module (correlation coefficient=0.6; p<0.0001) and the brown module (correlation coefficient=−0.76; p<0.0001) exhibited the strongest correlation with the ESRGs score (Figure 1E). A total of 3,425 genes in these two modules with MM>0.6 and GS>0.4 were selected as key module genes (Figure 1F and G).

### Identification of candidate genes and functional enrichment analysis

**Figure 2:**
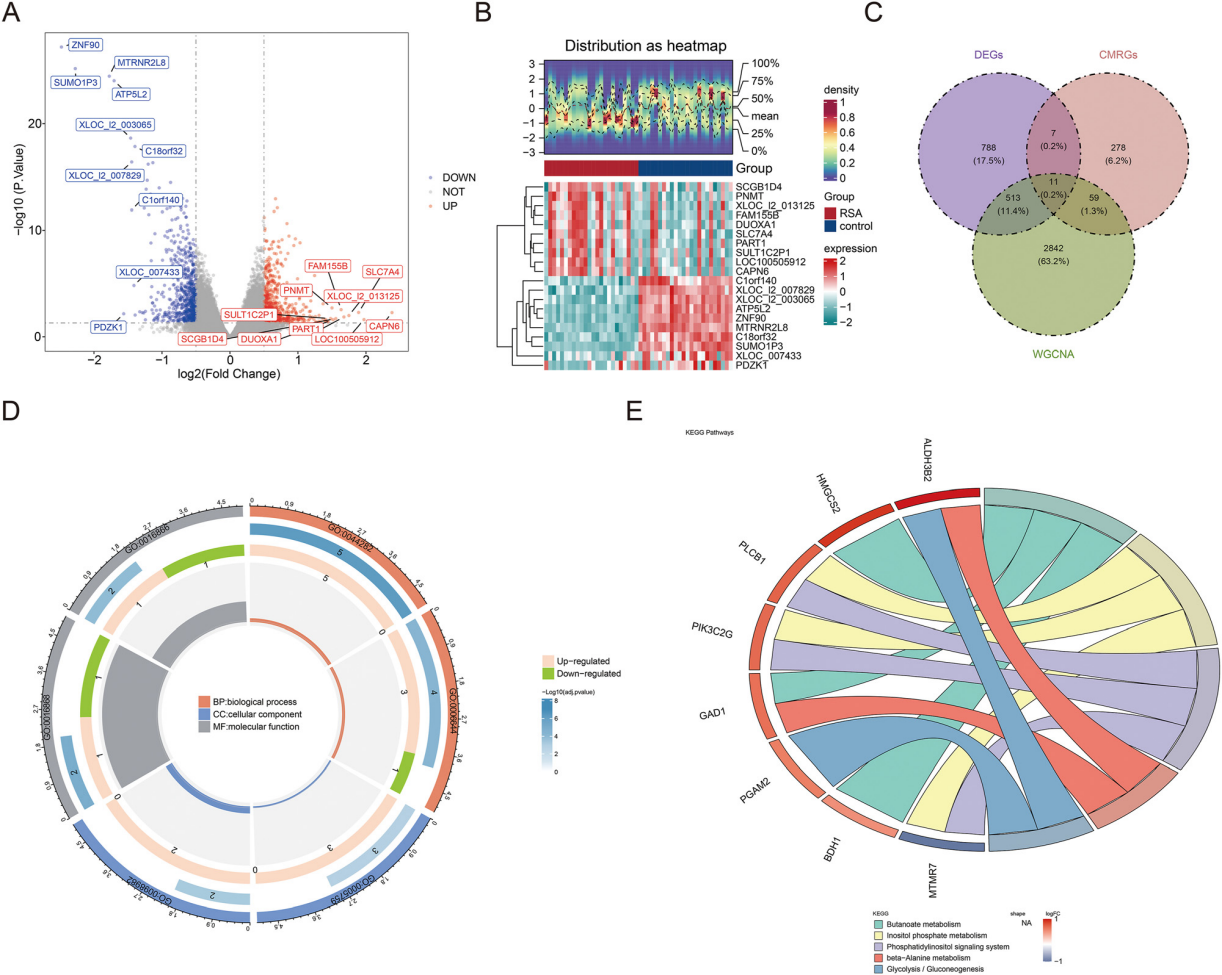
Identification of candidate genes and functional enrichment analysis. (A) The volcano plot illustrates the distribution of differentially expressed genes (DEGs) between RSA and control groups in the GSE165004 dataset (|log2 Fold Change|>0.5, p<0.05). Red dots indicate upregulated genes, and blue dots indicate downregulated genes. Labeled genes represent the top 10 DEGs in both subsets. (B) The heatmap displays the expression patterns of the top 10 upregulated and downregulated DEGs across RSA and control samples in the GSE165004 dataset. Red indicates higher expression, and blue indicates lower expression. (C) The Venn diagram shows the intersection of DEGs, ES-related key module genes from WGCNA, and CMRGs from the literature, with overlapping genes selected as candidate genes. (D) The circular plot summarizes the GO enrichment analysis of candidate genes in BP, CC, and MF categories. (E) The bar plot shows the top five enriched KEGG pathways associated with the candidate genes.

With the criteria of |log2Fold Change|>0.5 and p<0.05, we identified 1,319 DEGs between RSA and control samples from the GSE165004 dataset, including 631 upregulated and 688 downregulated (Figure 2A). The heatmap showed the expression of the top 10 upregulated and downregulated DEGs across the samples (Figure 2B). We then intersected these DEGs with the aforementioned key module genes from WGCNA and CMRGs from previous literature, yielding 11 overlapping genes as candidate genes (Figure 2C). Subsequently, functional enrichment analysis was conducted on these candidate genes to explore the potential biological functions and pathways associated with ES and CM that occur in RSA. GO annotation analysis revealed that the candidate genes were significantly enriched in 14 biological processes (BP), with the top five items involving small molecule catabolic processes, phospholipid metabolic processes, phosphatidylinositol metabolic processes, glucose metabolic process, and hexose metabolic process (Figure 2D, Supplementary Table S4). Moreover, they were significantly associated with 25 molecular functions (MF), including intramolecular transferase activity, and oxidoreductase activity, acting on the CH-OH group of donors, NAD or NADP as acceptor, and 2 cellular components (CC), mitochondrial matrix and GABA-ergic synapse (Figure 2D, Supplementary Table S4). Consistently, KEGG pathway analysis depicted that the candidate genes were markedly involved in several metabolism-related pathways, including butanoate metabolism, inositol phosphate metabolism, phosphatidylinositol signaling system, β-alanine metabolism, and the bidirectional pathway of glycolysis and gluconeogenesis (Figure 2E, Supplementary Table S5). These findings suggested a potential role for candidate genes in regulating a wide range of metabolic processes and functional pathways.

### Identification of BDH1 and PIK3C2G as key genes for RSA

**Figure 3:**
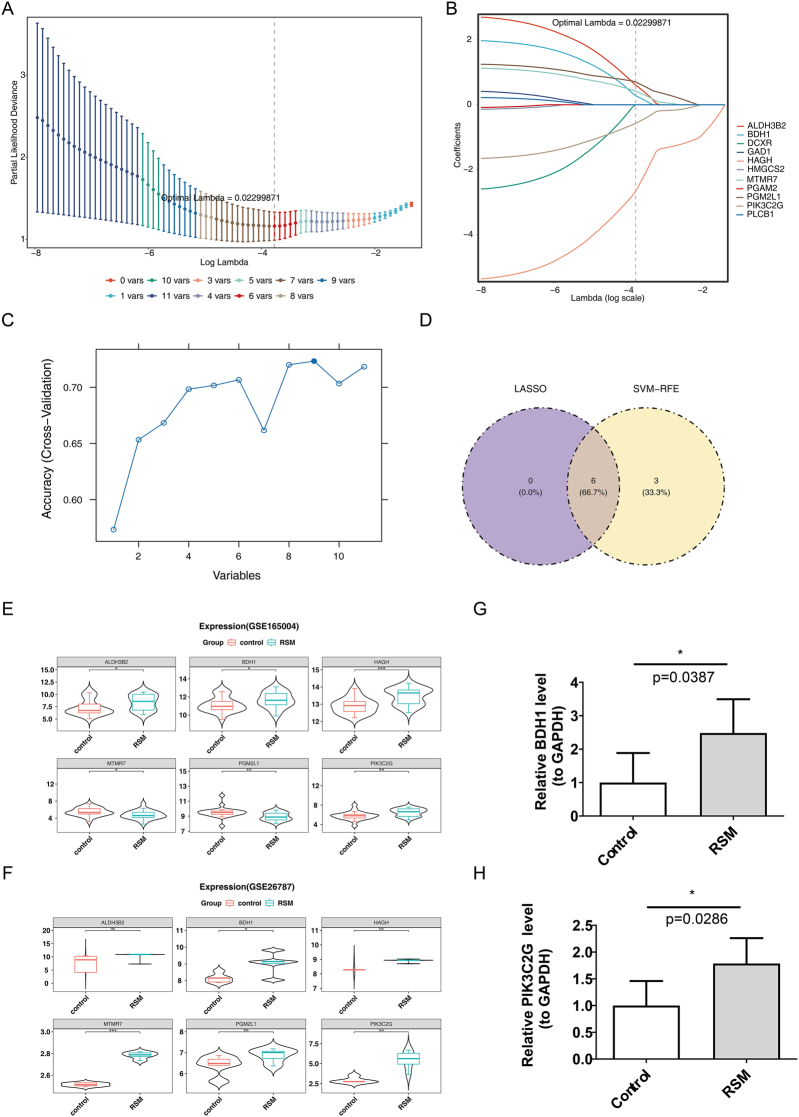
Identification of BDH1 and PIK3C2G as key genes for RSA. (A) Partial likelihood deviance plot for LASSO regression. The plot shows the partial likelihood deviance as a function of the log lambda values in the LASSO regression model. (B) Coefficient profile plot for LASSO regression. The coefficient profile plot illustrates the shrinkage of coefficients for each gene as the lambda value increases in the LASSO regression model. The optimal lambda value (0.02299871) was used to select six genes (ALDH3B2, BDH1, HAGH, MTMR7, PGM2L1, and PIK3C2G) with non-zero coefficients. (C) The accuracy plot shows the cross-validation accuracy of the SVM-RFE algorithm as a function of the number of variables (genes). The optimal number of genes (nine) was selected based on the highest accuracy. (D) The Venn diagram shows the overlap between the feature genes identified by LASSO regression and SVM-RFE. The violin plots display the expression levels of the six candidate genes (ALDH3B2, BDH1, HAGH, MTMR7, PGM2L1, and PIK3C2G) in RSA and control samples from the training dataset (GSE165004) (E) and the validation dataset (GSE26787) (F). *p<0.05, **p<0.01, ***p<0.001. ns, no significance. Bar graphs of RT-qPCR show the relative mRNA levels of BDH1 (G) and PIK32G (H) in endometrial tissue specimens from RSA patients and healthy controls.

The LASSO model achieved the lowest error rate at a λ value of 0.02299871 and screened six feature genes (Figure 3A and B), and the SVM-RFE algorithm selected nine feature genes (Figure 3C). The feature genes obtained from the two machine learning algorithms were intersected, yielding six overlapping genes: HAGH, PGM2L1, PIK3C2G, MTMR7, ALDH3B2, and BDH1 (Figure 3D). We then analyzed the expression patterns of these six genes. As displayed in Figure 3E and F, ALDH3B2, HAGH, and PGM2L1 showed no significantly differential expression between RSA control samples in the GSE26787 dataset, and MTMR7 showed opposite expression patterns in the GSE165004 and GSE26787 datasets, which were therefore excluded in subsequent analyses. On the contrary, BDH1 and PIK3C2G, which were significantly highly expressed in the RSA group in both datasets, were selected as key genes for further analysis (Figure 3E and F). RT-qPCR was carried out to validate BDH1 and PIK3C2G expression patterns in RSA. Consistent with the microarray results, we found that BDH1 and PIK3C2G expression levels in the RSA group were remarkably higher than those in the controls (Figure 3G and H).

### Analysis of immune cell infiltration and its association with key genes

**Figure 4:**
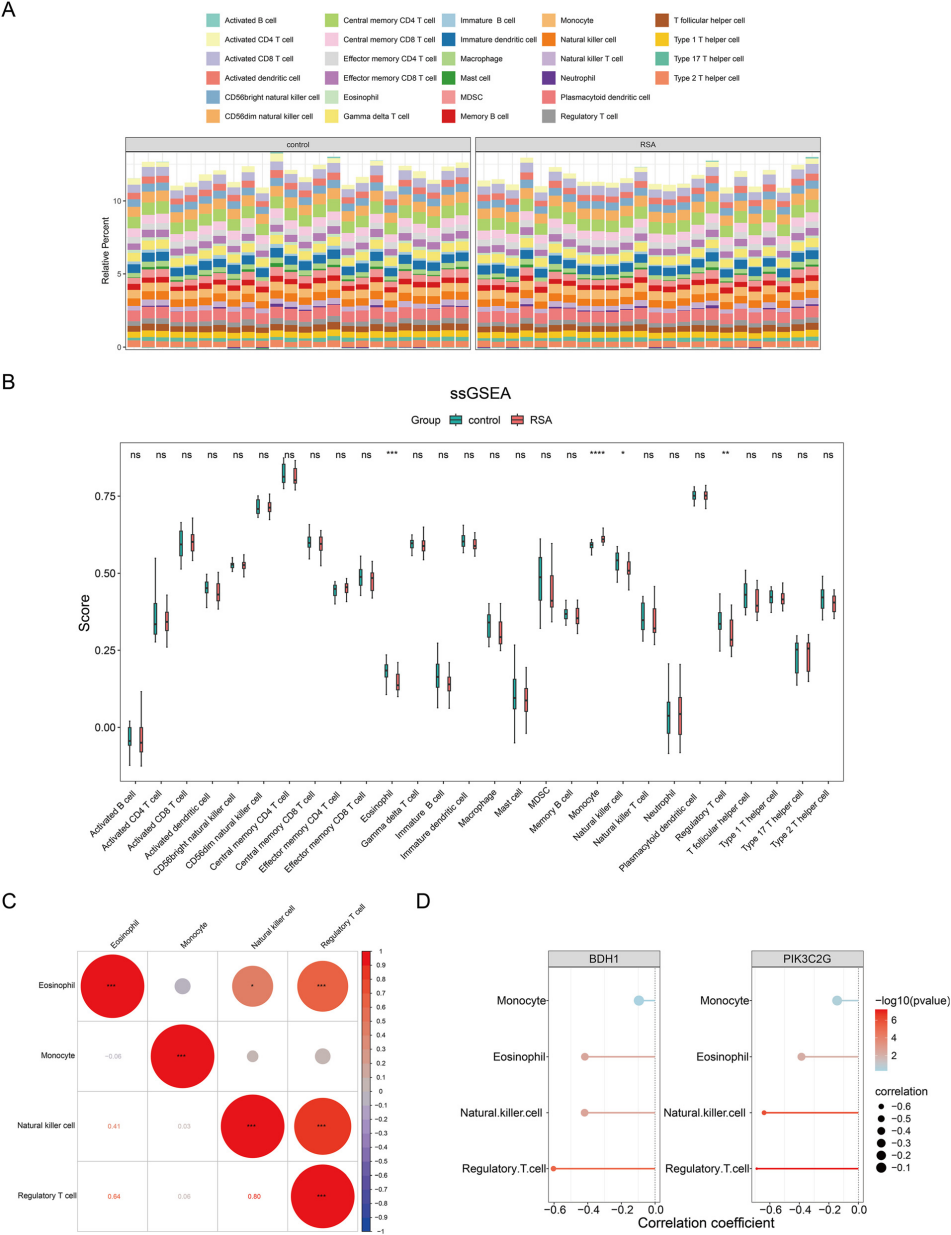
Analysis of immune cell infiltration and its association with key genes. (A) Boxplots display the relative abundance of 28 immune cell types in RSA and samples using ssGSEA. (B) Bar plots show differences in ssGSEA scores for 28 immune cell types between the RSA and control groups. *p<0.05, **p<0.01, ***p<0.001. ns, no significance. (C) The heatmap illustrates Spearman correlation coefficients between the four differentially infiltrated immune cell types (eosinophils, monocytes, natural killer cells, and regulatory T cells). (D) The scatter plots show the Spearman correlation between the expression levels of key genes (BDH1 and PIK3C2G) and the infiltration scores of eosinophils, monocytes, natural killer cells, and regulatory T cells.

To explore immune response mechanisms in RSA, the ssGSEA algorithm was utilized to estimate differences in immune infiltration between RSA patients and healthy fertile females. It was found that 28 types of immune cells were fairly evenly distributed in the samples (Figure 4A). Significant differences were observed in the abundance of four immune cell types between the RSA and control groups, including eosinophils, monocytes, natural killer cells, and regulatory T cells (Figure 4B). The interactions between these four immune cell types were assessed via Spearman correlation analysis. There was a significant negative correlation between natural killer cells and regulatory T cells. We also observed that eosinophils showed a significant positive correlation with both regulatory T cells and natural killer cells (Figure 4C). Notably, all these immune cell types, except monocytes, were negatively correlated with the two key genes, BDH1 and PIK3C2G (Figure 4D).

### GSEA and TF/drug-key gene network construction

**Figure 5:**
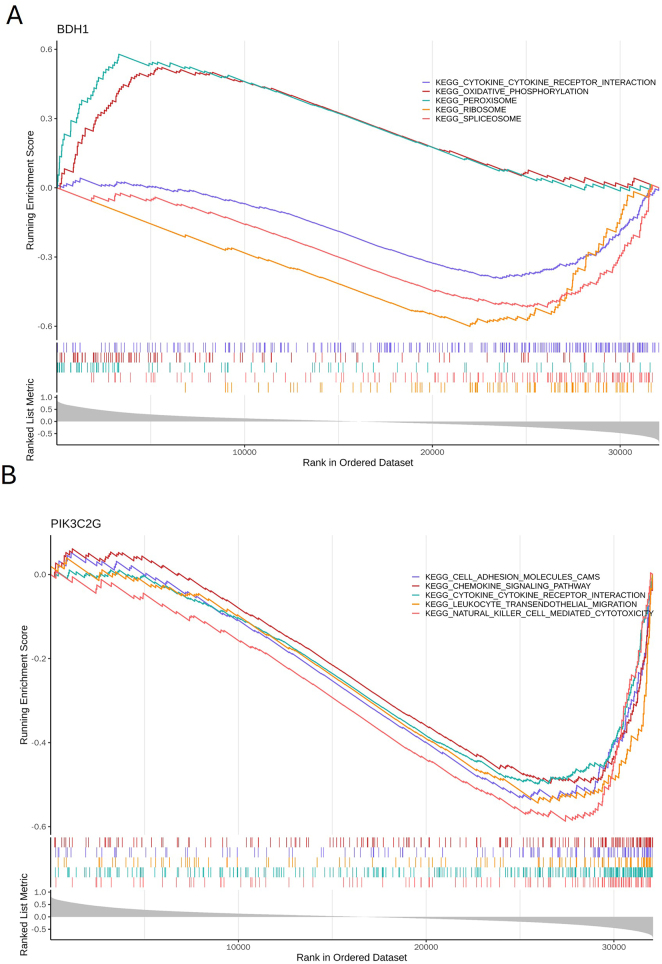
GSEA of BDH1 and PIK3C2G. The GSEA plot visualized the enrichment of KEGG pathways associated with BDH1 expression (A) and PIK3C2G expression (B). Adjusted p value <0.05.

**Figure 6:**
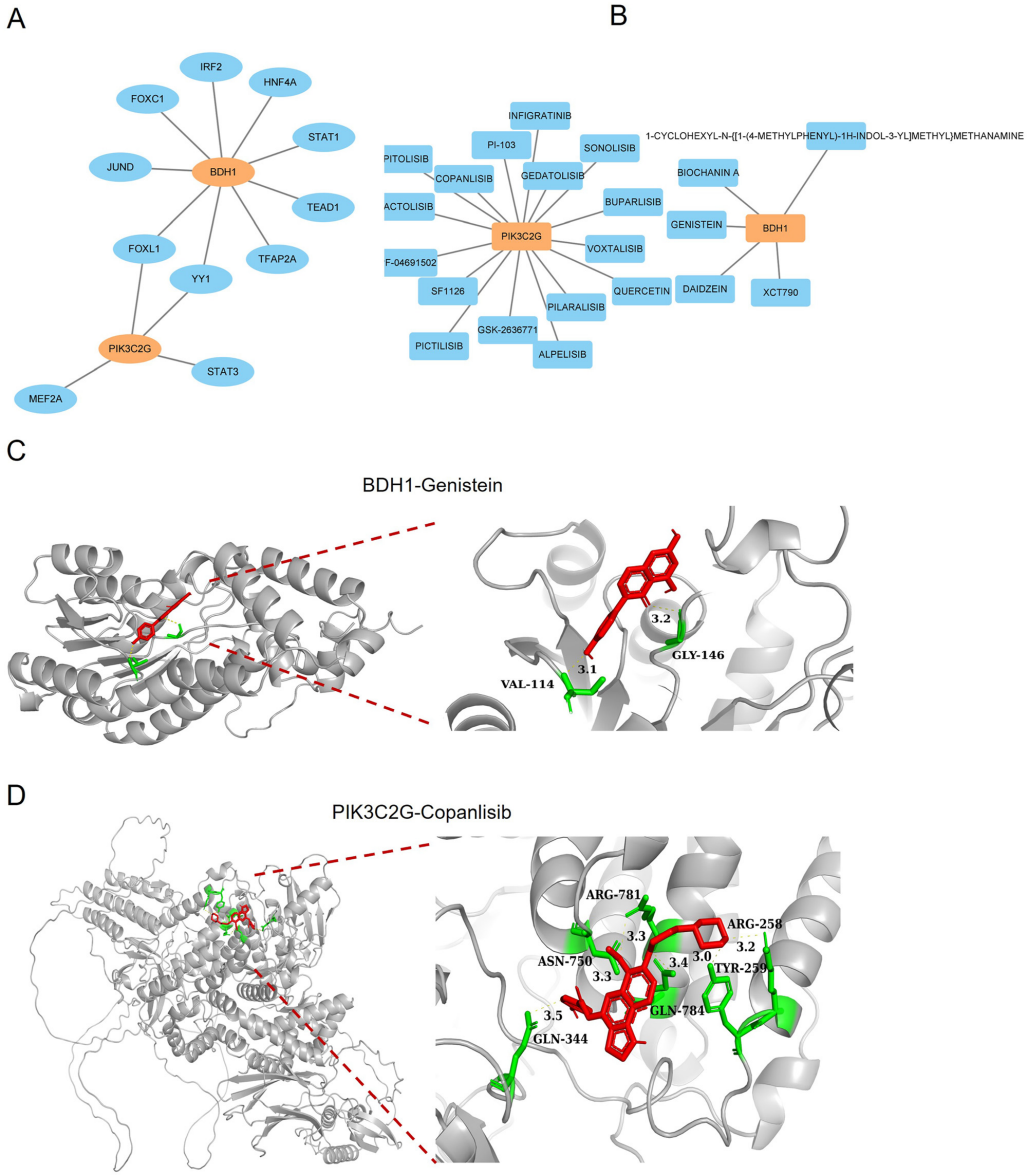
TF/drug-key gene network construction. (A) The network diagram displays transcription factors (TFs) that potentially regulate BDH1 and PIK3C2G. (B) the network diagram shows potential therapeutic drugs targeting BDH1 and PIK3C2G, as predicted by the Drug-Gene Interaction Database (DGIdb). Visualization of molecular docking analysis between BDH1 and genistein (C) or between PIK3C2G and copanlisib (D).

GSEA-KEGG pathway analysis was carried out for a deeper understanding of the molecular mechanisms of BDH1 and PIK3C2G. The results revealed that BDH1 was found to be enriched in 54 pathways, while PIK3C2G was found to be enriched in 75 pathways (Tables S6 and S7). Among the top five pathways associated with the two genes, only one pathway, namely, cytokine-cytokine receptor interaction, showed a downward trend in both (Figure 5A and B). Besides, our study predicted nine TFs targeting BDH1 and four TFs interacting with PIK3C2G, sharing two common TFs: FOXL1 and YY1 (Figure 6A). Moreover, a drug-gene interaction network was presented, encompassing five chemicals associated with BDH1 and 16 chemicals associated with PIK3C2G, with no common drug shared by the two genes (Figure 6B). Based on credibility, BDH1 with genistein and PIK3C2G with copanlisib were selected for molecular docking. The molecular docking results showed that the binding energies of BDH1 with genistein and PIK3C2G with copanlisib were −7.8 kcal/mol and −7.5 kcal/mol, respectively (Figure 6C and D), indicating the strong binding affinity between BDH1 and genistein and between PIK3C2G and copanlisib.

## Discussion

In the present study, we found that BDH1 and PIK3C2G are key genes at the intersection of ES and CM pathways in RSA. These genes were consistently upregulated in RSA samples compared to controls in both training and validation datasets, validated by RT-qPCR in clinical specimens. Furthermore, they showed negative correlations with specific immune cells and were enriched in the cytokine-cytokine receptor interaction pathway, with potential regulation by FOXL1 and YY1.

BDH1 (3-hydroxybutyrate dehydrogenase 1) is essential for maintaining energy balance and metabolic homeostasis, particularly in fatty acid, triglyceride, and ketone body metabolism [33]. In diabetic cardiomyopathy, reduced BDH1 expression can impair ketone utilization, exacerbating cardiac energy deficiency and dysfunction [34]. Moreover, BDH1 catalyzes the interconversion between β-hydroxybutyrate and acetoacetate [33], which can influence human embryonic viability and fetal development [35]. As a member of the phosphatidylinositol 3-kinase family, PIK3C2G regulates cell proliferation, tumorigenesis, migration, survival, and intracellular protein transport [36]. Previous studies have reported the association of PIK3C2G with several diseases, including liver cancer, colorectal cancer, and type 2 diabetes mellitus [36], [37], [38]. However, to our knowledge, no study has examined the involvement of BDH1 or PIK3C2G in RSA pathogenesis. Here, we confirmed the upregulation of BDH1 and PIK3C2G in endometrial/decidual tissues from RSA patients compared with controls, indicating the potential of these two genes in regulating RSA progression. Although the RT-qPCR validation was performed on bulk decidual tissue, which contains endothelial cells among other cell types, this approach lacks cell-type resolution. Thus, the observed upregulation of BDH1 and PIK3C2G supports differential expression at the maternal tissue level in RSA but does not directly confirm endothelial cell specificity or senescence status. The differences may arise in part from alterations in stromal or immune cell composition.

The immune system is critical in maintaining pregnancy and embryo implantation. Immune abnormalities may cause the mother’s body to perceive the embryo as a foreign entity, prompting an attack that ultimately results in the miscarriage [39]. An imbalance in immune cells, including both innate and adaptive immune cells, contributes to RSA progression [39]. Our study revealed that four immune cell types were differentially abundant between the RSA and control groups, including eosinophils, monocytes, natural killer cells, and regulatory T cells. Notably, both BDH1 and PIK3C2G were found to have a significantly negative correlation with eosinophils, natural killer cells, and regulatory T cells. Eosinophils secrete various cytokines that regulate the immune response, participate in the allergic response, and influence T-cell function [40]. Natural killer cells mediate immune tolerance during early pregnancy, ensuring normal fetal development [41]. Research indicates a notable shift in the distribution of natural killer cell subsets within the peripheral blood of RSA patients, potentially linked to pregnancy complications [42]. Over 60 % of women suffering from unexplained RSA during the 4 to 6-week gestational period exhibited a marked decline in peripheral natural killer cell levels in early pregnancy when compared to their pre-pregnancy state [43]. The primary function of regulatory T cells is to suppress excessive immune responses and maintain maternal-fetal immune tolerance [44]. If the inhibitory function is impaired, the maternal immune system may attack embryonic tissues, potentially triggering an immune rejection response, which is detrimental to the implantation and development of the embryo in the uterus, thereby leading to miscarriages [45]. Therefore, the upregulation of BDH1 and PIK3C2G may disrupt the immune balance at the maternal-fetal interface, thereby contributing to RSA. Immunomodulatory treatment has been shown to improve reproductive outcomes in women with RSA [46]. Further investigation is needed to determine the effects of BDH1 and PIK3C2G on the immune microenvironment and immune cell infiltration to help develop treatment strategies via immune modulation.

GSEA results showed that BDH1 and PIK3C2G are involved in multiple pathways, including cytokine-cytokine receptor interaction, oxidative phosphorylation, and chemokine signaling pathway, which are closely linked to immune inflammation. Significantly elevated levels of inflammatory cytokines, such as IL-6 and TNF-α, have been observed in the placental tissues of patients with RSA [47]. Previous evidence has indicated the inseparable relationship between obesity and CM disorders, especially insulin resistance [48]. Preliminary experiments revealed that endothelial cell homeostasis in the placenta was disturbed, and fetal vasculature was impaired in obese dams, accompanied by IL-6 upregulation. Additionally, elevated IL-6 levels can induce ES [49]. These indicate that CM disorders aggravate inflammation and ES, leading to placental dysfunction, ultimately contributing to pregnancy losses.

TFs are a group of proteins that bind to gene-specific sequences to modulate gene transcription. Multiple TFs play pivotal roles in RSA pathogenesis [50]. In the present study, to explore the potential molecular mechanism of the two key genes (BDH1 and PIK3C2G), we constructed TF-key gene networks and obtained several TF-BDH1/PIK3C2G pairs. Notably, transcription factors FOXL1 and YY1 are connected with both genes. As a multifunctional TF, YY1 can affect the development of the follicle, placenta, and embryo and has been identified as a key regulator in various reproductive diseases, including RSA [51]. Previous reports have shown FOXL1’s functional roles in multiple pathophysiological processes, such as lung fibroblast function and tumor cell aggressiveness [52], 53]. Nevertheless, there is no report on its role in RSA, highlighting the need for further investigation. Furthermore, we utilized key genes in combination with the DGIdb database to predict potential drugs for RSA therapy. Several drugs associated with BDH1 or PIK3C2G were predicted, especially genistein with BDH1 and copanlisib with PIK3C2G, providing potential directions for subsequent drug discovery and therapeutic strategies.

However, our study still has some limitations. First, the sample size in this study was relatively small, which may limit the generalizability of our findings. Future research will expand to large-scale multi-center cohorts and incorporate additional public datasets for cross-validation of our results. Second, although this study showed the predictive value for RSA, cell- or animal-based experimental validation is lacking. In future studies, we plan to perform gene knockout experiments in RSA-related cell lines, such as trophoblast and endometrial cells, as well as RSA animal models to investigate the *in vivo* effects of these genes on pregnancy outcomes. Moreover, all transcriptomic and RT-qPCR analyses were performed on bulk tissues, lacking cell-type resolution. Although the ssGSEA enrichment score of the ESRGs was used to identify correlated modules via WGCNA, this score in bulk tissue cannot directly demonstrate endothelial-cell senescence per se, as it may be influenced by shifts in cell-type proportions (e.g., endothelial cell content or immune cell infiltration) rather than genuine per-cell senescence activity. Thus, although BDH1 and PIK3C2G showed consistent upregulation, these findings support tissue-level associations with ES-related pathways and CM but cannot directly confirm endothelial cell specificity or senescence status. Additionally, the GEO datasets represent non-pregnant mid-secretory phase endometrial biopsies, whereas our validation used pregnant decidual tissues, which differ substantially in pregnancy state, cell composition, immune milieu, and metabolic context. However, the consistent upregulation of BDH1 and PIK3C2G across both tissue types supports the robustness of their association with RSA. Future studies employing single-cell RNA sequencing or spatial transcriptomics will be essential to confirm cell-type specificity and to further elucidate the mechanistic links to ES and CM.

In summary, this study identified two key genes, BDH1 and PIK3C2G, at the intersection of CM and ES pathways in RSA by utilizing various bioinformatics approaches. These two key genes are closely related to the development of RSA and are associated with multiple immune infiltrates. They may serve as potential targets for the diagnosis and treatment of RSA.

## Supplementary Material

Supplementary Material

Supplementary Material

Supplementary Material

Supplementary Material

Supplementary Material

Supplementary Material

Supplementary Material

Supplementary Material
