# Supplementary material for: Discovery and validation of key genes and potential mechanisms linked to endothelial cell senescence and carbohydrate metabolism in recurrent spontaneous abortion
Source: Open Med (Wars). 2026 Jun 24;21(1):20261464. doi: 10.1515/med-2026-1464 (PMC13293328; doi:10.1515/med-2026-1464)
Supplement: Supplementary file 3 — Supplementary Material [file j_med-2026-1464_suppl_003.doc]

**Table S1 The Primer Sequences of hub genes**

| Primer | Sequence | |
| --- | --- | --- |
| BDH1 F | GGTGGAGGGGCTTCCAGAAA | |
| BDH1 R | CAAAACCAAATGGGCAGGGG |  |
| PIK3C2G F | ATCAACCCCATTCTTCTAGCCA |  |
| PIK3C2G R | CCAGGAGAGTTCACGGCTTTT |  |
| Reference: GAPDH F | CGAAGGTGGAGTCAACGGATTT |  |
| Reference: GAPDH R | ATGGGTGGAATCATATTGGAAC |  |
