# Supplementary material for: Discovery and validation of key genes and potential mechanisms linked to endothelial cell senescence and carbohydrate metabolism in recurrent spontaneous abortion
Source: Open Med (Wars). 2026 Jun 24;21(1):20261464. doi: 10.1515/med-2026-1464 (PMC13293328; doi:10.1515/med-2026-1464)
Supplement: Supplementary file 8 — Supplementary Material [file j_med-2026-1464_suppl_008.docx]

**Supplementary Materials**

Table S1. The list of 102 ESRGs from previous literature.

Table S2. The list of 355 CMRGs from previous literature.

Table S3. Primer sequences used for RT-qPCR.

Table S4. GO analysis results of candidate genes.

Table S5. KEGG pathway analysis of candidate genes.

Table S6. The result of the GSEA-KEGG analysis for BDH1.

Table S7. The result of the GSEA-KEGG analysis for PIK3C2G.
